# Microbiological characteristics of a novel species most closely related to 'Bergeyella cardium' as a pathogen of infectious endocarditis

**DOI:** 10.1371/journal.pone.0191715

**Published:** 2018-01-25

**Authors:** Li-Na Guo, Ying Li, Po-Ren Hsueh, Peng Wang, Yu-Pei Zhao, Ying-Chun Xu

**Affiliations:** 1 Department of Clinical Laboratory, Peking Union Medical College Hospital, Peking Union Medical College and Chinese Academy of Medical Sciences, Beijing, China; 2 Departments of Laboratory Medicine and Internal Medicine, National Taiwan University Hospital, National Taiwan University College of Medicine, Taipei, Taiwan; 3 Department of General Surgery, Peking Union Medical College Hospital, Peking Union Medical College and Chinese Academy of Medical Sciences, Beijing, China; Beijing Institute of Microbiology and Epidemiology, CHINA

## Abstract

Infectious endocarditis (IE) can be caused by various pathogens, from dominating agents such as viridans group streptococci and staphylococci to rare species that are less virulent and not typically considered to be pathogens. In this study, we have isolated a novel species from a patient with problem of IE which was genetically most closely related to ‘Bergeyella cardium’, a causative pathogen of IE first reported in Korea in 2015 as a new species of the genus *Bergeyella*, with a similarity of 98.8% in 16S rRNA sequences. Microbiological characteristics, including morphology, biochemical identification and antimicrobial susceptibility profiling, of this novel species were determined. This fastidious Gram-negative bacillus could only be identified successfully by molecular sequencing analysis at present, and it exhibited low minimum inhibitory concentrations to the antibiotics tested except for aminoglycosides. Phylogeny analysis revealed this novel species clustered well with ‘B. cardium’ and other close species of genus *Bergeyella*.

## Introduction

The world in which human beings live is a huge reservoir of diverse microorganisms including bacteria, fungi and viruses. In recent decades, more and more emerging but less common pathogens have been isolated from clinical settings as the microbiology laboratory technologies have become more progressive. Furthermore, some bacteria that were previously known only as environmental microorganisms have been reported to cause human infections, which may be due partially to the increased virulence of these microbes and the impairment of human immune systems [1–3].

In the present study, we reported a case of infective endocarditis (IE) caused by a novel fastidious bacillus species belonging to the family *Flavobacteriaceae* which is phylogenetically closely related to ‘Bergeyella cardium’, a new species in genus *Bergeyella* described in 2015 but not validly named to date [4]. Moreover, the morphological characteristics and antimicrobial resistance profile of this novel species were described, and difficulties encountered in its identification and antimicrobial susceptibility testing process were also discussed. To our knowledge, this is the first intensive research study of this novel species.

## Methods

### Ethics statement

This study protocol was approved by the Human Research Ethics Committee at Peking Union Medical College Hospital (No. S-263), and written consent was obtained from the patient concerned.

### Clinical case

A 24-year-old male was admitted to Peking Union Medical College Hospital, Beijing, China, on 13 May 2015 because he had been experiencing intermittent fever with chills and fatigue for six months. Antibiotic therapy (lack of detailed information) prescribed by the community hospital was completed before he came to our hospital, but the symptoms persisted. On physical examinations, the patient was revealed to be healthy except for an unstable temperature with intermittent fever, hypotension [103/38 mmHg] and weight loss of 15 kg during the course of illness. Laboratory tests revealed that the patient had an increased erythrocyte sedimentation rate (ESR) of >140 mm/h and suspected anemia with Hb of 57 g/L. His symptoms were slightly alleviated after a blood transfusion with 2 units of condensed erythrocytes. Cardiac auscultation revealed consecutive murmurs in the second aortic valve area. Echocardiography was prescribed to determine the cause of the murmurs. Congenital heart disease (CHD) of patent ductus arteriosus (PDA) and IE were diagnosed with evidence of aortic regurgitation and multiple vegetations on the pulmonary valve and pulmonary artery wall. Based on the above evidence and the current physical condition, the patient was hospitalized in the cardiac surgery department on 26 May, and a cardiac surgical operation was performed to rectify the CHD and IE condition on 1 June 2015. A peripheral blood sample was sent to microbiology laboratory for blood culture testing on 26 May and was positive; the pathogen from the blood culture was revealed to be a novel species of the family *Flavobacteriaceae* on 6 June. Empirical treatment with ceftriaxone (IV, 2 g, qd) for 2 weeks during his hospitalization was prescribed. The patient recovered well after the operation and was discharged on 10 June 2015 with prolonged antibiotic treatment with ceftriaxone for 5 weeks. Since he was discharged, no significant clinical events have occurred during follow-up.

### Initial laboratory examinations

After sixty-eight hours of incubation, the blood culture (aerobic bottle) in a BACTEC 9000 system (Becton Dickinson, Sparks, MD) was positive and pathogen identification was carried out immediately. Briefly, approximately 1 ml of the positive blood culture was used to inoculate Columbia Blood agar, Chocolate agar and China Blue Lactose agar (Oxoid, Wesel, Germany) respectively, which were then incubated at 35±1°C with 5% CO_2_ supplement. Staining tests (Gram staining plus Acid fast staining or not), automated identification systems (Vitek 2 compact system, bioMérieux, France; BD phoenix 100 automated microbiology system, Becton, USA) and MALDI-TOF MS systems (Vitek MS v.2.0 system, bioMérieux, France; Bruker Biotyper v.3.1 system with the available database DB 5989, Bruker Daltonics, Germany) were employed to identify the positive colonies grew on the agar plates. 16S rRNA sequencing analysis with the universal primers of 27F and 1492R was performed for a precise species-level identification [5, 6]. No other specimens were sent for microbiological examination.

### Antimicrobial susceptibility testing (AST)

After the initial identification was completed, AST was not carried out immediately as usual due to the lack of a reference method in the Clinical and Laboratory Standards Institute (CLSI) document concerning the taxon to which this novel species belongs [7]. Minimum inhibitory concentrations (MICs) of certain supposedly effective antibiotics against this isolate were evaluated by the E-test method to determine its antimicrobial susceptibility profile when we were constructing this study. Briefly, a 1.0 McFarland turbidity inoculum suspension of this organism in saline was prepared through the direct colony suspension method. Columbia Blood agar plates were inoculated with a sterile cotton swab dipped into the suspension within 15 minutes using an automatic plate rotator. E-test strips (Bio Mérieux, France) were firmly applied to the surface of the inoculated agar plates. Those plates were placed in an incubator at 35±1°C with 5% CO_2_ supplementation. The MICs were read when distinct inhibition zones appeared on the inoculated plates.

### Phylogenetic analysis

The 16S rRNA sequences of this novel species and other closely related species in the family *Flavobacteriaceae* available in the GenBank database were retrieved for phylogenetic analysis with MEGA (Molecular Evolutionary Genetic Analysis, version 6.0) [8]. A phylogenetic tree was constructed using the Neighbor-Joining (NJ) method [9], with all positions containing gaps and missing data eliminated from the data set. The stability of the grouping was estimated by bootstrap analysis (1000 replications).

## Results

### Identification of the pathogen

The positive blood culture was detected on 29 May after sixty-eight hours of incubation, and colonies were harvested on 1 June after three days of sub-inoculation on Columbia Blood agar plates due to the slow growth property of this isolate. Initially, the isolate was identified as *Brevundimonas* spp. by the Vitek 2 compact system (bioMérieux, Marcy l’Etoile, France) with positive biochemical activities of lipase, phosphatase and arylamidase of amino acids, including Ala, Phe, Pro, Tyr, Glu, Gly, and Arg. The BD Phoenix 100 system, Vitek MS system and Bruker Biotyper MALDI-TOF MS system with official databases all failed to identify this isolate. 16S rRNA sequencing analysis revealed this strain should be identified as a novel species closest to ‘Bergeyella cardium’, with greatest pairwise similarity of 98.8% to ‘B. cardium’ strain 13-07^**T**^ (Accession No. KJ817166.1). The 16S rRNA sequence of this novel species was uploaded to the GenBank database with an Accession No. KY089082.1.

We have made in-house spectrum for this novel species with Bruker Biotyper MALDI-TOF MS system and good performance was achieved. The effectiveness of this in-house spectrum was quietly well since samples of this isolate could all be identified correctly with log scores of >2.300 while other clinical isolates (n = 589) including bacteria, yeasts and molds didn’t get any negative impact on the identification after affiliating this in-house spectrum with the Bruker official database (Data not shown).

### Morphological characteristics of this novel species

**Fig 1:**
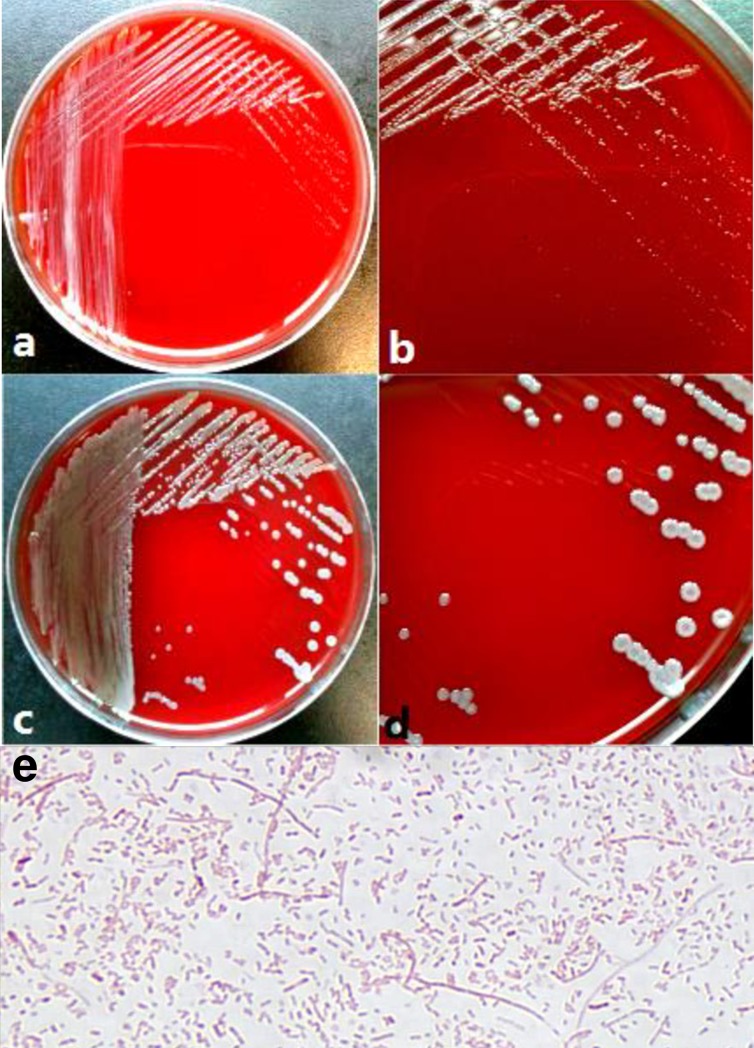
**Morphological characteristics of strain PU13217, a novel species, on Columbia Blood agar (a to d) and its Gram staining property (e).** Incubation conditions: **1a** and **1b**, 35°C + 5% CO_2_, 2 days; **1c** and **1d**, 35°C + 5% CO_2_, 5 days.

This fastidious microbe grew slowly on Columbia Blood agar at 35°C with 5% CO_2_ supplementation. After 2 to 3 days of incubation, small, moist, smooth, gray-white-colored colonies appeared on the agar plate without any pigment or hemolysis. After five days of incubation, the colonies became bigger, and slight concavity was detected in the middle of single colonys. This strain was also able to grow on Chocolate agar at 35°C with 5% CO_2_ supplementation, but formed smaller and whiter colonies than those on Columbia Blood agar. Growth without CO_2_ supplementation failed on both Blood agar and Chocolate agar. Mueller-Hinton agar and China Blue Lactose agar did not support growth regardless of the incubating temperature and CO_2_ supplementation. Gram staining revealed this strain to be a Gram-negative bacillus of irregularly shaped rods (Fig 1).

### Antimicrobiol resistance profile

As this fastidious bacillus isolate could not grow on Mueller-Hinton broth or Mueller-Hinton agar even with 5% sheep blood supplement, AST was ultimately performed on Columbia Blood agar plates using the E-test method. Antibiotics including penicillins, cephalosporins, carbapenems and aminoglycosides that are supposed to kill gram-negative bacilli effectively were evaluated [4,7]. MICs were read when distinct inhibition zones appeared on the second day (48 hours of incubation) and were re-measured on the third day. No significant difference was detected between the two sets of MIC results (Table 1). Despite the absence of criteria for interpreting the MICs of this novel species, this strain may be susceptible to most of the tested antibiotics except for aminoglycosides, as higher MICs of 24 μg/ml and 32 μg/ml against Amikacin (AK) and Tobramycin (TM), respectively, were found.

**Table 1:** Minimum inhibitory concentrations (MICs) of strain PU13217 to sixteen antimicrobial agents.

Agent	MICs (μg/ml) determined on 48 and 72 hours after incubation	
	48 hours	72 hours
Benzylpenicillin	0.006	0.006
Ampicillin	≤ 0.016	≤ 0.016
Cefuroxime	≤ 0.016	≤ 0.016
Cefaclor	0.032	0.032
Ceftazidime	≤ 0.016	≤ 0.016
Ceftriaxone	0.006	0.006
Cefepime	≤ 0.016	≤ 0.016
Imipenem	0.012	0.012
Ertapenem	0.023	0.023
Cefoperazone-sulbactam	≤ 0.016	≤ 0.016
Piperacillin-tazobactam	≤ 0.016	≤ 0.016
Ciprofloxacin	0.094	0.094
Moxifloxacin	0.125	0.125
Levofloxacin	0.19	0.19
Amikacin	24	24
Tobramycin	32	48

### Phylogenetic analysis

**Fig 2:**
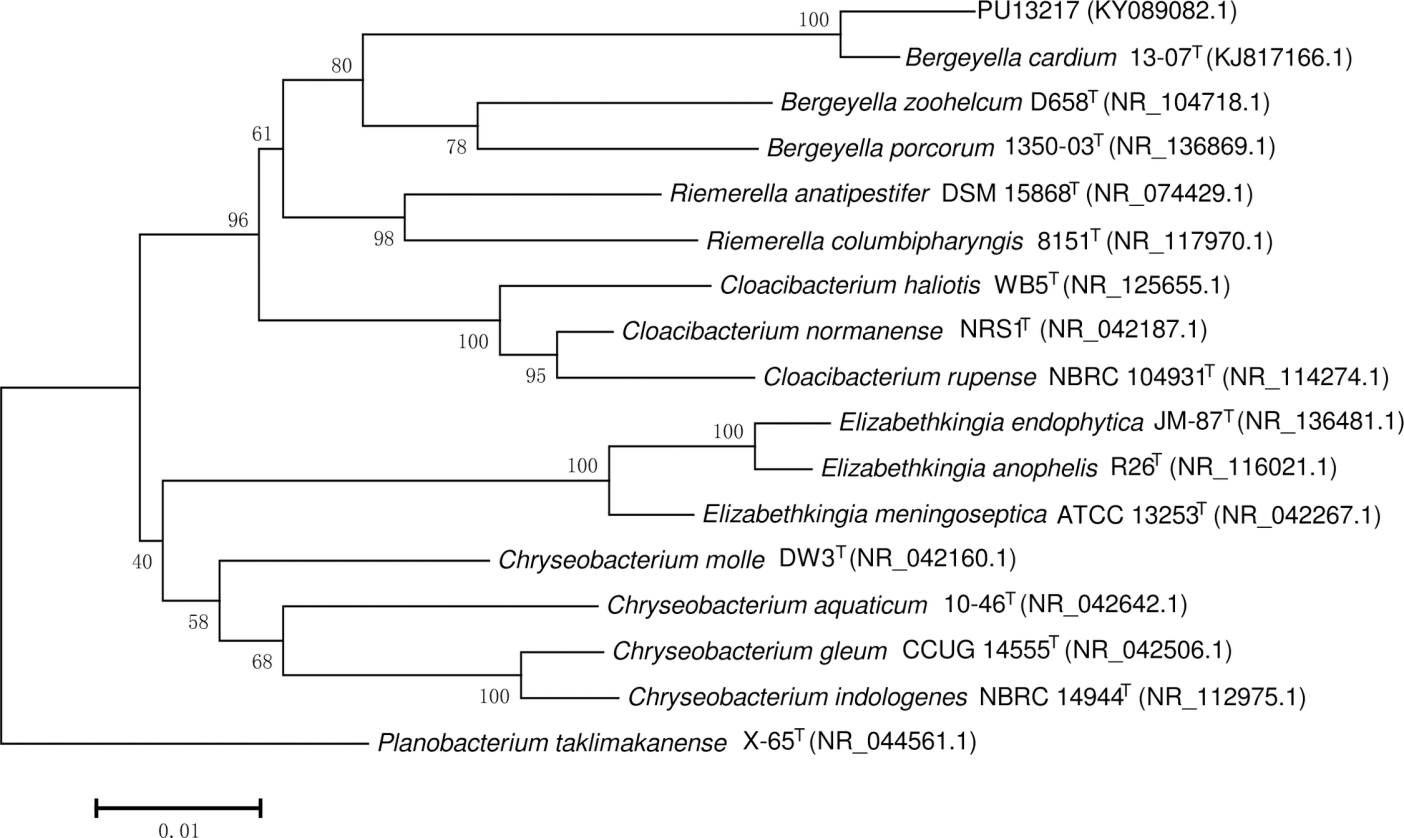
Phylogenetic tree based on the 16S rRNA sequences of strain PU13217, a novel species, and type strains of other closely related species. *Planobacterium taklimakanense* X-65 was used as the outgroup.

In the phylogenetic tree constructed with 16S rRNA sequences, this newly isolated species, represented by strain PU13217 in our study, clustered well with ‘B. cardium’ strain 13-07^T^ with a 100% bootstrap value and could be grouped into one clade with the other two species of the genus *Bergeyella*, *B*. *zoohelcum* and *B*. *porcorum* (Fig 2).

## Discussion

More diverse but rarely encountered microbes have been reported to be pathogens that can cause infectious endocarditis as the microbiology diagnostic technologies become more advanced. ‘Bergeyella cardium’, first isolated from two IE patients in Korea in 2015, described as a newly identified species in the *Bergeyella* genus even not validly named to date, is considered to be an IE pathogen. In this study, we isolated another novel species of bacillus from a patient suffering with IE that is most closely related to ‘B. cardium’. All the three patients had similar non-acute clinical symptoms and recovered well after treatment with antibiotics or additional surgical operations [4]. In our case, empirical antibacterial therapy with ceftriaxone was carried out once the diagnosis of IE was given after echocardiography examination. The diagnosis and treatment remained unchanged after this novel pathogen was identified from a positive blood culture of this patient, which could mainly be attributed to a poor understanding of this novel species, particularly its antibiotic susceptibility profile at that moment.

This novel fastidious Gram-negative bacillus cannot be identified by the current commercially available identification systems, including automatic ID systems and MALDI-TOF MS systems. 16S rRNA sequencing analysis was the only valid method for identifying this species, which was classified as a novel species closely related but not identical to ‘B. cardium’ as it shares 98.8% similarity with the 16S rRNA sequence of ‘B. cardium’ strain 13-07^T^ (Accession No. KJ817166.1) based on the criteria of the CLSI MM18-A document in which similarities of 97% to 98.9% to the 16S rRNA sequence of a type strain or validly named species indicates a novel species closely related to the compared species [6].

AST was performed using the E-test method when this study was prepared. Due to the slow growth of this fastidious bacillus, we arbitrarily adopted the Columbia Blood agar plate as the inoculating plate and inoculated with 1.0 McFarland inoculum suspension of this organism instead of the standard 0.5 McFarland inoculum to avoid a loss of potency of the E-test strips during the required longer incubation time. This strain was revealed to be susceptible to β-lactamase and quinolones but had higher MICs against aminoglycosides. Due to the lack of information about the previous antibiotic treatment the patient received before visiting our hospital, what’s more, aminoglycoside susceptibility testing was not performed for the two ‘B. cardium’ isolates in Korea, it's difficult to determine whether the aminoglycoside resistance suggested by higher MIC values of this strain was acquired from the prior medication this patient received or being intrinsically resistant to aminoglycosides which is a particular feature of family *Flavobacteriaceae* [10,11].

This novel species is closely related to ‘B. cardium’ both from the highly similar pathogenicities and closely phylogenetic relationship. As for the other species in genus *Bergeyella*, namely *B*. *zoohelcum* and *B*. *porcorum*, the only two validly named species in this genus to date, the similarities of this novel species with those two species in 16S rRNA sequences are fairly lower: 94.5% with *B*. *zoohelcum* strain D658^T^ (Accession No. NR_104718.1) and 93.4% with *B*. *porcorum* strain 1350-03^T^ (Accession No. NR_136869.1), respectively. *B*. *zoohelcum* is known to be part of the normal oral flora of cats, dogs and other animals and is associated with rare but severe human clinical diseases such as cellulitis, leg abscesses and septicemia after bites or scratches from cats and dogs [12,13]. *B*. *porcorum* was isolated in 2016 from a pig and hold an ambiguous relationship with pneumonia in pigs [14,15]. However, these four species clustered well into one clade with reasonable bootstrap values, which strongly suggests a possibility that the patient isolate represents a new species in the *Bergeyella* genus and so further studies should be done to investigate the close taxonomic relationships for taxa discussed here.

## Conclusions

We have described an IE case in China caused by a novel bacterial species most closely related to ‘Bergeyella cardium’. The microbiological characteristics of this causative agent, including its morphology, antimicrobial susceptibility profile and phylogeny, were discussed, which provides an intensive understanding of this novel species. The higher MICs of this isolate against aminoglycosides highlights the importance of accurate identification. 16S rRNA sequencing analysis is the only effective method to identify this novel species to date while MALDI-TOF MS will be helpful after a well-identified in-house spectrum is created.

## References

[1] Johnson PT, Ostfeld RS, Keesing F. Frontiers in research on biodiversity and disease. Ecol Lett. 2015; 18(10):1119–33. http://doi.org/10.1111/ele.12479 . 26261049 10.1111/ele.12479 PMC4860816

[2] Kesson AM, Kakakios A. Immunocompromised children: conditions and infectious agents. Paediatr Respir Rev. 2007; 8(3):231–9. http://doi.org/10.1016/j.prrv.2007.07.005 . 17868921 10.1016/j.prrv.2007.07.005

[3] Walsh TJ, Groll A, Hiemenz J, Fleming R, Roilides E, Anaissie E. Infections due to emerging and uncommon medically important fungal pathogens. Clin Microbiol Infect. 2004; 10 Suppl 1:48–66. 14748802 10.1111/j.1470-9465.2004.00839.x

[4] Sohn KM, Huh K, Baek JY, Kim YS, Kang CI, Peck KR, et al A new causative bacteria of infective endocarditis, *Bergeyella cardium* sp. Nov. Diagn Microbiol Infect Dis. 2015; 81(3):213–6. http://doi.org/10.1016/j.diagmicrobio.2014.12.001 . 25544000 10.1016/j.diagmicrobio.2014.12.001

[5] Hayashi H, Sakamoto M, Kitahara M, Benno Y. Diversity of the Clostridium coccoides group in human fecal microbiota as determined by 16S rRNA gene library. FEMS Microbiol Lett. 2006; 257(2):202–7. http://doi.org/10.1111/j.1574-6968.2006.00171.x . 16553854 10.1111/j.1574-6968.2006.00171.x

[6] Clinical and Laboratory Standards Institute. 2008 Interpretive criteria for identification of bacteria and fungi by DNA target sequencing: guideline. CLSI document MM18-A. Clinical and Laboratory Standards Institute, Wayne, PA.

[7] Clinical and Laboratory Standards Institute. 2017 Performance Standards for Antimicrobial Susceptibility Testing; Twenty-seventh informational supplement. CLSI document M100-S27. Clinical and Laboratory Standards Institute, Wayne, PA.

[8] Sohpal VK, Dey A, Singh A. MEGA biocentric software for sequence and phylogenetic analysis: a review. Int J Bioinform Res Appl. 2010; 6(3):230–40. http://doi.org/10.1504/IJBRA.2010.034072 . 20615832 10.1504/IJBRA.2010.034072

[9] Saitou N, Nei M. The neighbor-joining method: a new method for reconstructing phylogenetic trees. Mol Biol Evol. 1987; 4(4):406–25. doi: 10.1093/oxfordjournals.molbev.a040454 . 3447015 10.1093/oxfordjournals.molbev.a040454

[10] Fass RJ, Barnishan J. In vitro susceptibilities of nonfermentative gram-negative bacilli other than *Pseudomonas aeruginosa* to 32 antimicrobial agents. Rev Infect Dis. 1980; 2(6):841–53. . 7012987 10.1093/clinids/2.6.841

[11] Chang JC, Hsueh PR, Wu JJ, Ho SW, Hsieh WC, Luh KT. Antimicrobial susceptibility of flavobacteria as determined by agar dilution and disk diffusion methods. Antimicrob Agents Chemother. 1997; 41(6):1301–6. . 9174188 10.1128/aac.41.6.1301 PMC163904

[12] Shukla SK, Paustian DL, Stockwell PJ, Morey RE, Jordan JG, Levett PN, et al Isolation of a fastidious *Bergeyella* species associated with cellulitis after a cat bite and a phylogenetic comparison with *Bergeyella zoohelcum* strains. J Clin Microbiol. 2004; 42(1):290–3. doi: 10.1128/JCM.42.1.290-293.2004 . 14715767 10.1128/JCM.42.1.290-293.2004 PMC321680

[13] Yi J, Humphries R, Doerr L, Jerris RC, Westblade LF. *Bergeyella zoohelcum* associated with abscess and cellulitis after a dog bite. Pediatr Infect Dis J. 2016; 35(2):214–6. http://doi.org/10.1097/INF.0000000000000971 . 26535880 10.1097/INF.0000000000000971

[14] Zamora L, Domínguez L, Fernández-Garayzábal JF, Vela AI. *Bergeyella porcorum* sp. nov., isolated from pigs. Syst Appl Microbiol. 2016; 39(3):160–3. http://doi.org/10.1016/j.syapm.2016.03.006 . 27039167 10.1016/j.syapm.2016.03.006

[15] Oren A, Garrity GM. List of new names and new combinations previously effectively, but not validly published. Int J Syst Evol Microbiol. 2016; 66(7):2463–6. http://doi.org/10.1099/ijsem.0.001149 27530111 10.1099/ijsem.0.001149

